# The effect of transcutaneous neuromuscular electrical stimulation on laryngeal vestibule closure timing in swallowing

**DOI:** 10.1186/s12901-018-0054-3

**Published:** 2018-05-08

**Authors:** Christopher R. Watts, Matthew J. Dumican

**Affiliations:** Davies School Communication Sciences & Disorders, Texas Christian University, TCU Box 297450, Fort Worth, TX 76129 USA

**Keywords:** Swallowing, Deglutition, Deglutition disorders, Neuromuscular electrical stimulation

## Abstract

**Background:**

The purpose of this study was to investigate the effect of transcutaneous neuromuscular electrical stimulation (NMES) on the timing of laryngeal vestibule closure during the pharyngeal stage of swallowing in healthy adults. The theoretical framework proposed that NMES applied to these muscles would present a perturbation to laryngeal vestibular closure reaction time (the amount of time for the laryngeal vestibule to close once the swallowing reflex has been triggered) by providing an antagonistic force to the direction of vestibule closure.

**Methods:**

Nine healthy adults (2 males, 7 females) received ten consecutive stimulations applied to the submandibular hyolaryngeal muscles while performing dry swallows. Laryngeal vestibule closure reaction time (LVCrt) and the laryngeal vestibule closure duration (LVCd) were measured from videoflouroscopic images pre-stimulation and post-stimulation.

**Results:**

Results indicated a significant effect of stimulation on LVCrt but not LVCd. LVCrt was significantly reduced (timing was faster) during swallows immediately after stimulation compared to pre-stimulation.

**Conclusions:**

Findings from this study support the supposition that laryngeal muscles respond to perturbations via adaptation learning, which might be used for rehabilitation of neuromuscular swallowing impairment. This pilot study supports the need for further research.

## Background

Neuromuscular electrical stimulation (NMES) is used by allied health professionals as a treatment modality for neuromotor impairments including muscle weakness, muscle atrophy, and decreased range of motion [1–3]. In populations served by speech-language pathologists, these impairments are often associated with dysphagia caused by etiologies such as stroke and degenerative disease. The clinical research literature reporting outcomes for the effects of NMES on swallowing function is growing, as are the stimulation devices and NMES clinical protocol options available to professionals. The physiological adaptations secondary to NMES application for muscles involved during swallowing, specifically muscle activation during the pharyngeal stage, are less clear. If clinicians are to utilize NMES as a valid treatment modality, it is critical that they have knowledge of the physiological principles underlying its application and be able to apply those principles to impaired swallowing physiology.

Clinical goals when utilizing NMES often include strengthening, increasing tone (to reduce atrophy), or increasing range of motion in a target muscle. In order to increase muscular strength and tone, treatments often overload (stress) a muscle by providing a resistance to contraction force. When a resistance is applied against contraction, the muscle is stressed and over time the body will respond by adapting to the stress. One way muscles adapt to the stress of an overload or resistance against contraction is to hypertrophy. Muscle hypertrophy results from the muscle cells gaining volume. Regular application of resistance against contraction will also result in adaption of the neural drive to muscle. Neural drive adaptation results in a greater number of motor units recruited during a contraction. Some NMES protocols incorporate the concept of progressive resistance by setting an initial duty cycle to a smaller ratio of off to on time (i.e., 1:5) for a period of time (i.e., a week) and then increasing the ratio progressively over a subsequent period [4]. Progressive resistance may facilitate strength adaptation in muscle groups. By manipulating the relationship of the stimulation intensity and duty cycle, the clinician can take advantage of progressive resistance using NMES to facilitate adaptation in the targeted swallowing muscles.

Research has provided evidence that voluntary contraction with stimulation may cause activity in the central nervous system (CNS) which is different than when muscles are stimulated without voluntary contraction (i.e., stimulation only). Doeltgen et al. found that corticobulbar motor evoked potentials (an indirect measurement of CNS motor activity) were greater when NMES was applied to the submandibular muscles during volitional contraction compared to NMES applied to the same muscles when they were at rest [5]. The increased motor excitability of the CNS after NMES stimulation was present up to 60 min after treatment. This study suggested that NMES applied to the submandibular muscles during voluntary contraction resulted in changes to the corticobulbar pathways controlling those muscles. Specifically, NMES increased the “excitability” of the corticobulbar pathways that cause the submandibular muscles to contract. Given that reduced corticobulbar excitability might underlie many cases of post-stroke dysphagia, this finding provides support for further investigations which study the effect of submandibular NMES on neuromotor swallowing physiology. Based on the findings of Doeltgen et al., such studies should elicit swallows while an individual is receiving stimulation during swallowing trials, rather than stimulation without active contraction of the swallowing muscles.

NMES can also be used to facilitate motor adaptation learning through a process of perturbations to muscle contraction. When perturbations are introduced to a contraction, such as an added resistance or a force that moves structures in an antagonistic direction, the nervous system can adapt to the perturbation by recalibrating the motor programs associated with the intended movement [6]. It has been shown that motor adaptation learning can occur after brief exercise sets, and the learning effects last beyond the treatment phase [7]. This has implications for the application of NMES to impaired swallowing physiology. For example, if dysphagia is associated with impaired hyolaryngeal excursion and/or laryngeal closure, perturbations to the muscles which elevate the laryngeal complex or close the laryngeal vestibule might cause adaptations to that perturbation through a recalibrated motor response which improves laryngeal movement during the pharyngeal stage of swallowing. Humbert et al. have demonstrated this effect in healthy normals. Their experiment applied 25 consistent (as opposed to random) perturbations to hyolaryngeal excursion through NMES applied to the laryngeal depressors, presenting an antagonistic pull to the muscles which elevate the larynx while swallowing 5 ml of water [8]. Measurements of peak hyolaryngeal elevation increased after the perturbation trials, suggesting adaption motor learning occurred after only a brief training phase.

Our laboratory has consistently observed the following phenomena during visual inspection of videoflouroscopic images of individuals receiving NMES to the submandibular muscles: (a) the hyoid bone is moved in a superior and anterior direction [this effect has also been reported by Kim & Han, who demonstrated that NMES applied to the submandibular muscles resulted in vertical and anterior excursion of the hyoid by 9.6 mm and 1.9 mm, respectively [9], (b) the larynx is pulled in a superior direction, and (c) the laryngeal vestibule widens in the anterior dimension. The purpose of the present study was to apply the theory of adaptation learning via the introduction of consistent perturbations to laryngeal closure during swallowing using NMES. We hypothesized that NMES to the submandibular muscles would present a perturbation to laryngeal vestibule closure reaction time (e.g., the time it takes for the laryngeal vestibule to close once the swallowing reflex has been triggered), if the stimulation was applied during the act of swallowing. We also measured the total duration of laryngeal closure (e.g., the total duration the vestibule remains closed during the pharyngeal stage) to determine if perturbations affected the overall duration of laryngeal contraction during the pharyngeal phase of swallowing.

## Methods

### Participants

Nine healthy participants (2 male, 7 female) were recruited to participate in this study. Inclusion criteria for participation were: (a) no history of swallowing impairment, (3) no history of neurological disease or stroke, (3) less than 65 years of age, and (4) no self-reports of current swallowing problems. In addition, because electrical stimulation to the anterior neck would be used, pregnancy, pacemakers, or other implanted neurosensory stimulation devices were exclusionary factors.

### Equipment

Surface NMES was applied using an Ampcare ES™ electrical stimulator (Ampcare, LLC: Fort Worth, TX) and bilateral transcutaneous E-series electrodes (Ampcare, LLC: Fort Worth, TX). The E-series electrodes were triangular in shape and designed to fit within the submental region of the neck, superior to the hyoid bone. The electrodes were pre-gelled with self-adhering material on the inner surface allowing for direct fixation to the skin. Participant swallows were video recorded during videofluoroscopy. Temporal measures were obtained from video recordings using VDSC Video Editor software (Flash-Integro, LLC). This software allowed for advancement through the recordings at approximately 30 frames per second.

### Procedures

All procedures utilized in this study were approved by a university Institutional Review Board (# 1709–027-1710). After consenting procedures, bilateral electrodes were placed over the left and right submental muscles using the mental protuberance of the mandible as an anterior reference point and the thyroid notch as an inferior reference point for each participant. An orthotic posture device (Restorative Posture Device/RPD; Ampcare, LLC: Fort Worth, TX) was placed on the participant to support their neck in alignment with the cervical spine. This device ensured consistent positioning of head and neck posture of each participant for application of NMES. The Ampcare ES™ electrical stimulator was set as follows for each stimulation trial:

- A pulse rate of 30 Hz was chosen to produce muscle contraction needed for small muscle groups without over-fatiguing. This setting was chosen since the suprahyoid musculature are much smaller than traditional muscles found in the limbs and torso.
- An adjustable pulse width between 50 μsec and 250 μsec was used with the Ampcare ES unit. This allowed the investigator an option to select the most comfortable parameter for the participant based on the strength duration curve (e.g., low pulse width with a higher intensity or a high pulse width with a lower intensity). All stimulations were initiated at a pulse width of 50 μsec. Longer pulse widths yield deeper penetration of the stimulation current, which may create a pain or discomfort that counteracts the benefit of NMES. Starting at a lower pulse width allowed for elicitation of the most efficient muscle contraction with the least amount of discomfort.
- The amplitude or intensity was adjusted between 0 to 100 milliamps (mA) as needed to elicit a motor response (in this study, an NMES motor response was defined as excursion of hyoid). This range is within that of previously published literature and has been determined to be safe and potentially clinically effective. The participant was instructed to notify the examiner if they were unable to tolerate any level of stimulation. Participants were instructed to keep their mouths closed as the neck muscles began to tighten.
- An on-ramp of 1 s was used to aid in comfort of the stimulation. The on-ramp allowed the participant to adjust to the current more gradually before receiving the maximum stimulation amplitude. There was no off ramp.
- A 1:3 duty cycle of 5 s of stimulation on time and 15 s of stimulation off time was used for all stimulations during the study.
- Symmetrical biphasic waveforms were utilized.

Once electrodes were fixated to the skin, the stimulation parameters (specified above) of the Ampcare ES™ stimulator were adjusted to determine settings for the most effective motor response that the participant could comfortably tolerate. This procedure mirrored clinical applications of NMES in dysphagic patients. When these settings were established, each participant then produced dry swallows (e.g., swallowing saliva) in the following conditions:

1. Three pre-stimulation swallows (no NMES applied)
2. Ten swallows while NMES was applied, using the parameters described above
3. Three post-stimulation swallows (no NMES applied)

Dry swallows were utilized due to the supposition that NMES applied to the submandibular muscles will widen the laryngeal vestibule (and potentially expose the larynx to a greater risk of penetration with larger bolus trials). The ten swallows with stimulation served as the perturbation trials. Auditory cueing was provided by the examiner to trigger each participant swallow. In-between swallows and only when stimulation was off, participants were offered a sip of water to promote surface hydration of their oral and pharyngeal cavities. Each swallow trial was video recorded for later playback and analysis.

### Analyses

Two dependent variables were acquired from videoflouroscopic recordings: (a) laryngeal vestibule closure reaction time (LVCrt) and (b) duration of laryngeal vestibule closure (LVCd). LVCrt represented the temporal duration between the time point of the video recording corresponding to initiation of sustained superior movement of the arytenoids towards the laryngeal surface of the epiglottis, to the time point where the arytenoids contacted the epiglottis AND the supraglottal air space within the vestibule was completely sealed. LVCd represented the temporal duration between the endpoint of the LVCrt measure and the initiation of arytenoid descent from the epiglottis, verified by reappearance of the supraglottal air space. Both LVCrt and LVCd have been previously reported in existing literature [10]. Both measurements were calculated in milliseconds (ms), and obtained from the pre-stimulation and post-stimulation swallows. LVCrt and LVCd were averaged across the three swallows for each participant.

Two separate Wilcoxon Signed Ranks tests were applied to the LVCrt and LVCd data, respectively, representing a comparison of repeated pre-stimulation measurements to post-stimulation measurements. As this was an initial study in this line of investigation, the alpha level was held constant at 0.05 to maintain adequate statistical power given the low sample size. Measurement reliability was assessed by a second examiner (2nd author) who re-measured 30% the total swallows. The second examiner was blind to swallowing condition (pre-stimulation, post-stimulation). Correlational analyses via Pearson product moment correlations were applied to the initial and reliability measures to assess the degree of relationship.

## Results

**Fig. 1:**
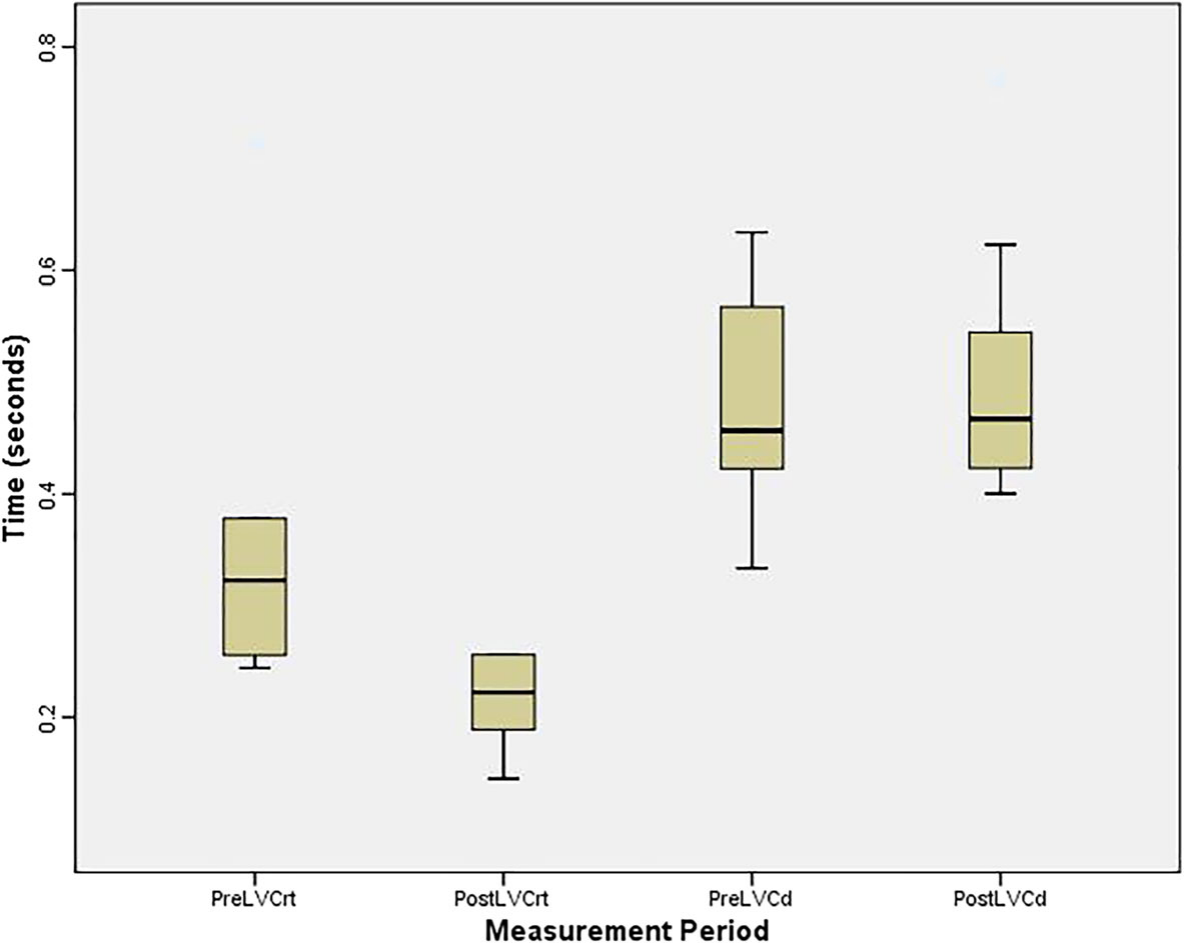
Box and Whisker plot illustrating the median (horizontal line within box), interquartile range (shaded area within box), and highest/lowest measures within 1.5× the interquartile range (upper and lower whiskers) for the dependent variables at the pre-stimulation and post-stimulation measurement periods

Table 1 presents mean LVCrt and LVCd data, averaged across the three pre-stimulation and post-stimulation trials for each participant. For LVCrt measurements, the data trend suggested that laryngeal vestibule closure occurred more quickly after the ten stimulation perturbations compared to before the perturbations. This trend was consistent for all nine participants. For LVCd measurements, the data trend suggested that a majority of participants (six) manifested longer vestibule closure after the stimulation perturbations, but two manifested shorter post-stimulation LVCd measures and one participant did not change. Figure 1 displays the raw data in graphic form, illustrating the pre-stimulation to post-stimulation changes in both dependent variables. Measurement reliability via correlational analyses was found to be very strong for both LVCrt (*r* = 0.99) and LVCd (*r* = 0.98) measures.

**Table 1:** Means (and standard deviation - sd) in seconds of the dependent variables, averaged for each participant across three swallows per measurement

Participant	Pre-Stimulation LVCrt	Post-Stimulation LVCrt	Pre-Stimulation LVCd	Post-Stimulation LVCd
1	0.71	0.18	0.57	0.62
2	0.32	0.26	0.46	0.51
3	0.24	0.22	0.45	0.46
4	0.37	0.21	0.63	0.54
5	0.38	0.26	0.58	0.42
6	0.38	0.26	0.38	0.40
7	0.24	0.14	0.42	0.42
8	0.31	0.22	0.33	0.47
9	0.26	0.19	0.57	0.77
MEAN (sd)	0.36 (0.14)	0.22 (0.04)	0.49 (0.10)	0.51 (0.12)

*LVCrt* laryngeal vestibule closure reaction time, *LVCd* laryngeal vestibule closure duration

Wilcoxon Signed Ranks tests were applied to LVCrt and LVCd measures separately. Results indicated a statistically significant difference in pre-stimulation and post-stimulation LVCrt measurements (z = − 2.67, *p* = 0.008). The effect size for this difference was large (*d* = 1.36). However, there was no statistically significant difference in pre-stimulation to post-stimulation LVCd measurements. The raw data indicated that LVCrt pre-stimulation durations of participant 1 were substantially longer than other participants. In order to determine if this influenced the statistical analyses, this participant’s LVCrt measures were removed from the data set and the Wilcoxon test was reapplied. The results remained statistically significant (*p* = 0.012) with a large effect size (*d* = 1.76).

## Discussion

The purpose of this study was to investigate the effect of perturbations, applied using NMES to the submandibular muscles, on timing of laryngeal vestibule closure (both reaction time to full closure and the total duration of closure) during swallowing to determine if adaptation learning occurred immediately after ten perturbation trials. The guiding theory was that NMES applied to the submandibular hyolaryngeal elevator muscles widens the laryngeal vestibule, which presents a resistance (and thus perturbation) to the muscular contractions that close the supraglottic laryngeal space during the pharyngeal stage of swallowing. Results supported the hypothesis, in that LVCrt was significantly shortened post-stimulation compared to pre-stimulation. The findings indicated that ten perturbation trials (stimulation to the submandibular muscles during “dry” swallow attempts) influenced the motor patterning in the pharyngeal stage within a short time frame, resulting in greater speed of laryngeal vestibule closure immediately after the stimulation trials.

The results of this study associated with LVCrt are aligned with the error-based learning of hyolaryngeal activity demonstrated by Humbert et al. and Anderson et al. [11, 12]. Those protocols utilized NMES applied to the infrahyoid muscles in order to elicit a perturbation to hyolaryngeal range of motion using either intermittent or masked stimulation conditions. By applying a resistance to hyolaryngeal excursion (stimulation of the infrahyoid muscles resists hyolaryngeal elevation), the authors hypothesized that NMES could elicit adaption learning through adjustments in the motor patterning of the pharyngeal stage through error-based motor learning. Their hypotheses were confirmed in a number of stimulation conditions (e.g., early versus later perturbation trials; masked versus unmasked trials). Both studies also demonstrated a short-term adaptation effect for temporal measures. Anderson et al., reported significant perturbation effects for temporal measures of duration to maximum hyoid elevation [12]. Humbert et al., using a measure corresponding to LVCrt in this study, found significant perturbation effects on the duration to laryngeal closure during 20 stimulations [11].

**Fig. 2:**
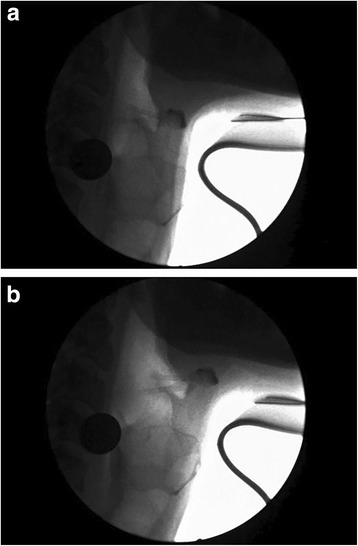
**a** & **b** Videoflouroscopic still frames showing the supraglottic air space and hyoid position prior to stimulation (2a) and during stimulation (2b) of the submandibular muscles. In 2b, the supraglottic air space has increased in volume due to an anterior pull emanating from tissue movement elicited by the NMES. When an individual swallows while the stimulation is on, this anterior pull on the laryngeal vestibule acts as a resistance to contraction, providing a perturbation to the movement

The change from pre-to-post stimulation LVCrt reported in this study most likely reflects short-term adaptations to the motor patterning of muscles responsible for sealing the supraglottic laryngeal air space. Figure 2a and b illustrate the effect that NMES applied to the submandibular muscles has on the air space within the laryngeal vestibule. With stimulation, the vestibular air space volume is increased in the anterior-posterior dimension (lateral dimensions could not be assessed in this plane of view). When swallowing during stimulation, the individual must contract against the antagonist resistance. Accordingly, neuromuscular control will be modified from trial-to-trial as a result of error feedback through sensory pathways in order to seal the supraglottic space [6]. Results from this study demonstrated this phenomenon to effect neuromuscular control by decreasing closure time. However, measures of LVCd were not significantly increased. The reason for this lack of response in the duration of laryngeal closure is unclear, as stimulation was on during the entire swallowing muscle contraction. It is possible that study limitations did not allow enough power to measure a potential difference, and future studies are needed to further investigate both LVCd and LVCrt in larger samples.

Guedes et al. have also demonstrated adaption learning influencing LVCrt using a volitional laryngeal vestibule closure maneuver, which incorporated resistance to relaxation of laryngeal muscles during the pharyngeal stage of swallowing (i.e., similar to a Mendelsohn maneuver) [10]. As in the present study, the authors reported effects of LVCrt during natural swallowing in healthy participants immediately after a training period of 20 volitional swallows using the maneuver. While exercises that incorporate perturbations via resistance (applied using NMES or volitional contractions) to facilitate laryngeal vestibule closure speed need to be studied for longer post-perturbation effects and in populations with dysphagia, their potential impact on swallowing rehabilitation is substantial. For example, hypokinesia of laryngeal movements resulting in penetration/aspiration is one of the most common manifestations of dysphagia in Parkinson’s disease (PD) [13, 14]. Rehabilitative exercises which facilitate adaptation learning through the application of perturbations to effectively accelerate laryngeal vestibule closure could thus facilitate swallowing safety and improve quality of life in a substantial number of individuals.

It has been previously demonstrated that NMES applied to the submandibular muscles (but not the thyrohyoid or other laryngeal depressors) effects anterior and superior displacement of the hyolaryngeal complex [9]. Inspection of Fig. 2a and b also reveals that stimulation, in this particular participant, can have the effect of displacing the hyoid bone and larynx in a similar direction. Impaired hyolaryngeal excursion underlies dysphagia in many treatment-seeking populations, including PD and post-stroke patients. In theory, NMES applied to the submandibular muscles when paired with swallowing exercises may have two different but complimentary effects for rehabilitating impaired hyolaryngeal function in swallowing. It could act as a perturbation to laryngeal vestibule closure, as demonstrated in this study, and also act as a facilitative modality for hyolaryngeal excursion by facilitating contraction of the submandibular muscles. By facilitating contraction in deconditioned or hypofunctional submandibular muscles, NMES may be able to induce adaption through muscle hypertrophy, contraction strength (via recruitment of motor neuron pools), and range of motion. This supposition has been partially supported by recent research. Toyama et al. compared submandibular NMES along with traditional therapy (Mendelsohn, thermal-tactile stimulation, and tongue exercises) to traditional therapy alone in groups of patients with dysphagia related to reduced hyolaryngeal excursion. The NMES + traditional therapy group exhibited greater post-treatment excursion of the hyoid along greater improvement in ratings of swallow function from videoflouroscopic studies [15]. Another recent study has also demonstrated that NMES to the submandibular muscles significantly improved swallow function post-stroke dysphagia [4]. Further clinical research is necessary to validate these findings.

## Conclusions

This study found significant immediate effects of NMES applied to the submandibular muscles on LVCrt during swallowing in healthy non-dysphagic volunteers. LVCrt was significantly faster during swallows immediately after NMES application compared to pre-stimulation. However the total duration of closure during the pharyngeal stage of swallowing was not affected by stimulation. Findings from this study add to existing evidence which has shown that the laryngeal muscles respond to perturbations via adaptation learning. Further research is needed to validate these findings, and to test if NMES applied as a perturbation to laryngeal vestibule closure has an effect on rehabilitating impaired swallowing secondary to a treatment dose. If adaptation learning can be facilitated with NMES perturbations during swallowing exercises, this modality might be considered as an option for rehabilitation of neuromuscular swallowing impairment. This pilot study supports the need for further research.

## Limitations of the study

A number of methodological limitations necessitate guarded generalizations from this study. The data set reported represents a small sample size (*n* = 9), and the design lacked a control group (i.e., no stimulation). While this is consistent with previous research on adaptation learning through perturbations to anterior neck muscles, future studies should include larger samples in an effort to replicate these findings. The participant sample utilized was healthy and less than 65 years of age. The effectiveness of NMES perturbations in older and dysphagic populations will also need to be validated in future research. Additionally, the effect of adaptation learning through perturbations was investigated at the immediate post-stimulation time frame. The goal of physical rehabilitation is to induce long-term training effects. Methodologies which investigate long-term effects of more prolonged training periods (e.g., across days, weeks, and months) will be a necessary next step for validation of the NMES perturbation effect on laryngeal vestibule closure reaction time.

## Abbreviations

CNS: Central nervious system
LVCd: Laryngeal vestibule closure duration
LVCrt: Laryngeal vestibule closure reaction time
NMES: Neuromuscular electrical stimulation
